# Evaluation of the accuracy of shoe fitting in older people using three-dimensional foot scanning

**DOI:** 10.1186/1757-1146-7-3

**Published:** 2014-01-23

**Authors:** Hylton B Menz, Maria Auhl, Sonja Ristevski, Nicoletta Frescos, Shannon E Munteanu

**Affiliations:** 1 Lower Extremity and Gait Studies Program, Faculty of Health Sciences, La Trobe University, Bundoora 3086, VIC, Australia; 2 Department of Podiatry, Faculty of Health Sciences, La Trobe University, Bundoora 3086, VIC, Australia

**Keywords:** Foot, Aged, Shoes, Anthropometry

## Abstract

**Background:**

Ill-fitting footwear is a common problem in older people. The objective of this study was to determine the accuracy of shoe fitting in older people by comparing the dimensions of allocated shoes to foot dimensions obtained with a three-dimensional (3D) scanner.

**Methods:**

The shoe sizes of 56 older people were determined with the Brannock device^®^, and weightbearing foot scans were obtained with the FotoScan 3D scanner (Precision 3D Ltd, Weston-super-mare, UK). Participants were provided with a pair of shoes (Dr Comfort^®^, Vista, CA, USA), available in three width fittings (medium, wide and extra wide). The dimensions (length, ball width and ball girth) of the allocated shoes were documented according to the last measurements provided by the manufacturer. Mean differences between last dimensions and foot dimensions obtained with the 3D scanner were calculated to provide an indication of shoe fitting accuracy. Participants were also asked to report their perception of shoe fit and comfort, using 100 mm visual analogue scales (VAS).

**Results:**

Shoe size ranged from US size 7 to 14 for men and 5.5 to 11 for women. The allocated shoes were significantly longer than the foot (mean 23.6 mm, 95% confidence interval [CI] 22.1 to 25.2; *t*_55_ = 30.3, *p* < 0.001), however there were no significant differences in relation to ball width (mean 1.4 mm, 95% CI −0.1 to 2.9 mm; *t*_55_ = 1.9, *p* = 0.066) or ball girth (mean −0.7 mm, 95% CI −6.1 to 4.8 mm; *t*_55_ = −0.2, *p* = 0.810). Participants reported favourable perceptions of shoe fit (mean VAS = 90.7 mm, 95% CI 88.4 to 93.1 mm) and comfort (mean VAS = 88.4 mm, 95% CI 85.0 to 91.8 mm).

**Conclusion:**

Shoe size selection using the Brannock device® resulted in the allocation of shoes with last dimensions that were well matched to the dimensions of the foot. Participants also considered the shoes to be well fitted and comfortable. Older people with disabling foot pain can therefore be dispensed with appropriately-fitted shoes using this technique, provided that the style and materials used are suitable and extra width fittings are available.

## Background

Footwear plays an important role in protecting the foot from extremes of temperature, moisture and mechanical trauma. However, since the development and widespread popularity of fashion footwear in the 17^th^ century, the functional aspect of footwear has largely been supplanted by the requirements of fashion. It has been suggested that shoe selection may be primarily based on aesthetic considerations, many of which are incompatible with the optimal function of the foot [1]. This is of particular concern in older people, as studies have shown that between 26 and 50% of older people wear shoes that are either too short or too narrow [2-5]. Several factors may be responsible for this, including fashion influences (particularly in older women [6,7]), not measuring foot dimensions when purchasing shoes [8], or the limited availability of commercially-available, low-cost footwear that adequately caters for the broader older foot [9,10].

There is growing evidence of an association between wearing shoes that are too small and the development of foot problems in older people. In a sample of 176 retirement village residents aged 62 to 96 years, Menz and Morris [11] found that wearing shoes substantially narrower than the foot was associated with corns on the toes, hallux valgus deformity and foot pain, whereas wearing shoes shorter than the foot was associated with lesser toe deformity. Similarly, in an ambulatory population of 213 people aged 60 to 80 years, Chaiwanichsiri et al. [3] reported that those who wore shoes that were too narrow were twice as likely to report foot pain. Most recently, a survey of 399 community-dwelling people aged over 60 years revealed that 61% of women and 30% of men reported foot pain when wearing shoes (most commonly in the forefoot and toes), and that women with foot pain exhibited a broader forefoot than those without pain [12].

Given the high prevalence of ill-fitting footwear in older people and its relationship to foot problems, accurate fitting of shoes in this age-group is an important consideration. Therefore, as part of a larger randomised controlled trial assessing the effectiveness of off-the-shelf footwear in reducing foot pain in Australian Department of Veterans’ Affairs (DVA) recipients not eligible for medical grade footwear [13], this study was undertaken to evaluate whether the fitting protocol employed in the trial resulted in the appropriate selection of shoe sizes. To do this, we compared the last dimensions of the shoes allocated to each participant based on an assessment using the Brannock device® [14] to corresponding foot dimensions obtained with a high resolution 3D foot scanner, and documented participants’ perceptions of the fit and comfort of the shoes.

## Methods

### Study design

This study was undertaken as part of a larger randomised controlled trial, the details of which have been published previously [13]. Briefly, the trial is a two-group randomised controlled trial design with a 16-week follow-up period, with participants randomly allocated to either a “usual care” control group or the intervention group. Both the control and intervention groups continued to receive usual podiatry care for the study period. This typically involved regular (every 6 to 8 weeks) toenail maintenance and scalpel debridement of keratotic lesions (corns and calluses). In addition, the intervention group was provided with off-the-shelf footwear at the baseline assessment, and data obtained from this group form the basis of the current study.

### Participants

Participants residing in Melbourne, Victoria, Australia were recruited from the DVA database between October 2012 and May 2013. To be eligible to be included in the study, veterans needed to: (i) be aged 65 years or over; (ii) be a current DVA Gold Card client (eligible for treatment of all their health care needs covered by the DVA) but not eligible for medical grade footwear; (iii) have received podiatry treatment on at least three occasions in the past five years; (iv) have disabling foot pain, using the case definition of the Manchester Foot Pain and Disability Index (MFPDI) [15] proposed by Roddy et al. [16]; (v) have persistent foot pain, defined as foot pain present for at least 12 weeks, and; (vi) be capable of understanding the English language in verbal and written form. Veterans were deemed ineligible for inclusion if they: (i) were currently residing in a high level care residential aged care facility; (ii) had diabetes and a history of foot ulceration (or current foot ulceration) or diabetic peripheral neuropathy (diagnosed with the 5.07 Semmes-Weinstein monofilament, using the International Working Group on the Diabetic Foot protocol [17]); (iii) had a neurodegenerative disorder (e.g. Parkinson’s disease); (iv) had a lower limb or partial foot amputation (although single toe amputations will be permitted); (v) had been prescribed contoured foot orthoses within the past 3 months; (vi) were currently wearing the intervention footwear, or; (vii) had cognitive impairment (defined as a score of <7 on the Short Portable Mental Status Questionnaire [18]).

The Australian DVA Human Research Ethics Committee provided ethical approval (approval number E012/005[5.1]) and the La Trobe University Human Ethics Committee formally accepted this approval (E012/004). All participants provided written informed consent prior to enrolment.

### Demographic information, medical history, foot disorders and footwear

Age, sex, height (cm), weight (kg) and body mass index (kg/m^2^) were documented for each participant. The medical history of participants was obtained via a structured questionnaire pertaining to the prevalence of major medical conditions and current medications. Foot pain duration and the presence of common foot disorders were documented using a standardised clinical assessment [19]. Style of footwear most commonly worn was documented using the 14-category ‘shoe type’ item of the *Footwear Assessment Tool*, which has been shown to have high intra- and inter-rater reliability (kappa values of 1.00 and 0.98, respectively) [20].

### Shoe fitting procedure

**Figure 1:**
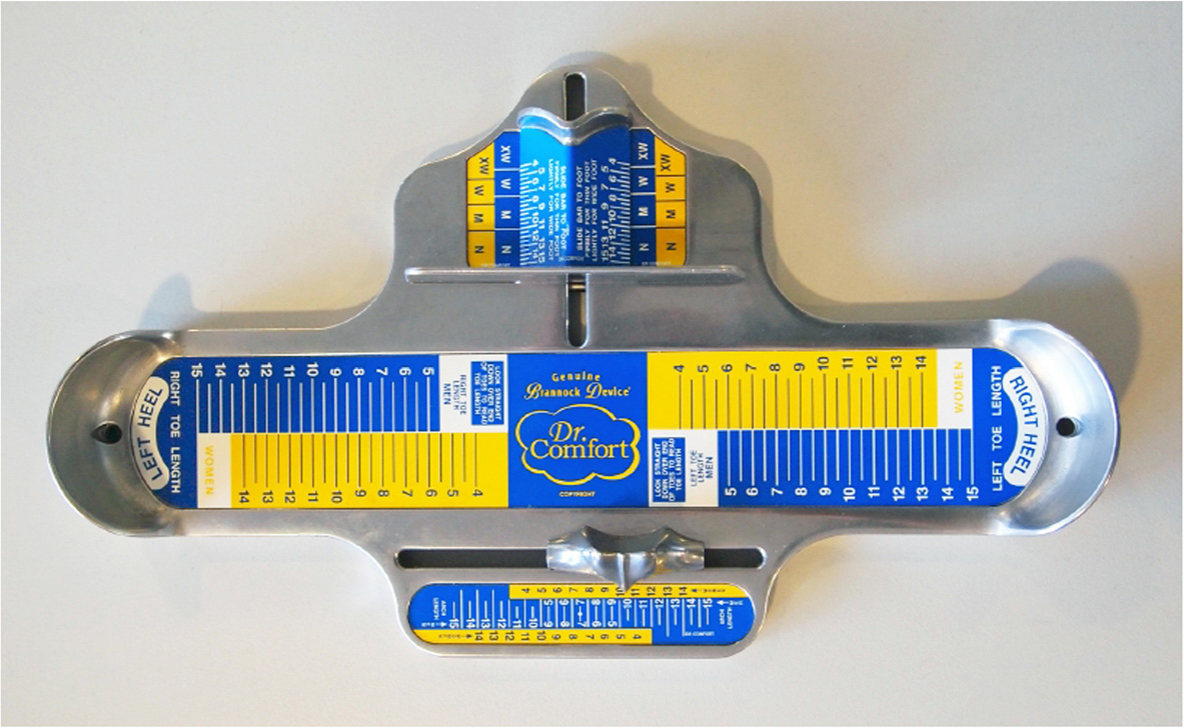
The Brannock device®.

Two trained research assistants (MA and SR) determined each participant’s shoe size with a Brannock device® [14] labelled with Dr Comfort® sizings using a standardised procedure [21] (Figure 1). Participants were instructed to stand relaxed, looking forward with their feet approximately shoulder width apart and their knees slightly flexed, and equal weight on each foot. The Brannock device® was placed under their foot, and with the heel placed securely in the device against the back of the heel cup, the length of the foot to the longest toe was determined and documented as the heel-to-toe measurement. Using the slide measure, the widest part of the foot (at the bisection of the medial aspect of the first metatarsophalangeal joint) was located and documented as the heel-to-ball measurement. An average of one heel-to-toe measurement and one heel-to-ball measurement was taken to determine the ball width by moving the slide measure in to contact with the lateral aspect of the foot. This process was then repeated for the other foot and documented as the ‘Brannock device®-determined size’. In the case of a discrepancy between right and left sizes, the longer (and/or wider) size combination was selected.

**Figure 2:**
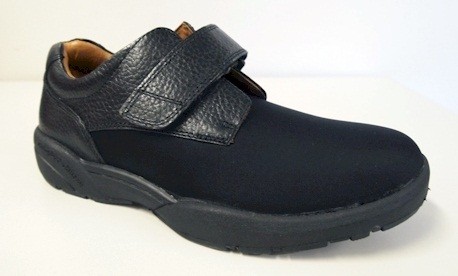
**Dr Comfort® footwear used in the study for men (*Brian *** **style).**

**Figure 3:**
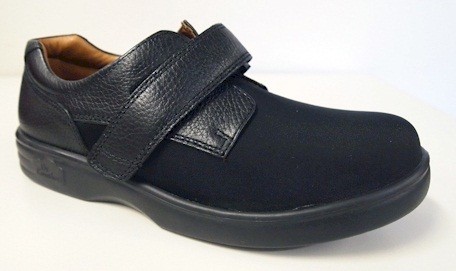
**Dr Comfort® footwear used in the study for women (*Annie *** **style).**

**Figure 4:**
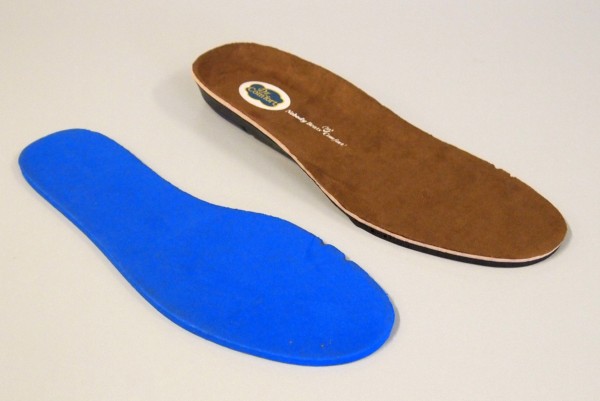
**The two insoles used.** Left: flat, 4 mm foam insole. Right: cushioning insole with a contoured heel cup.

**Figure 5:**
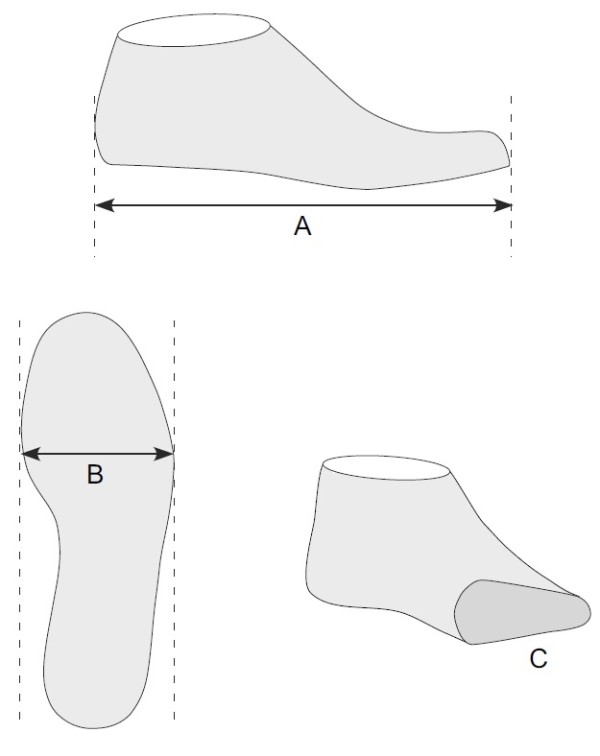
**Last measurements obtained from the footwear manufacturer. A**: length, **B**: ball width, **C**: ball girth.

The shoes used in the study were manufactured by Dr Comfort® (Vista, CA, USA). Men received the *Brian* style and women received the *Annie* style (see Figures 2 and 3). Both styles were available in three width fittings (medium, wide, extra wide) and featured a stretchable Lycra® (elastane) upper with Velcro® closure. These shoe styles were selected as they meet all commonly-used criteria for appropriate footwear (such as a low heel, appropriate fixation and adequate depth to accommodate toe deformities) [20,22-25]. The shoes came with a choice of two removable insoles: (i) a flat, 4 mm foam insole and; (ii) a cushioning insole with a contoured heel cup, 7 mm thick under the forefoot and 15 mm thick under the heel (Figure 4). Due to sex differences in foot dimensions (and therefore the dimensions of the lasts the shoes are constructed from) the *Brian* style had a relatively broader fit than the *Annie* style. The manufacturers provided the dimensions of the lasts (length, ball width and ball girth) for each style in each of the length and width fitting combinations (Figure 5).

A pair of shoes in the Brannock device®-determined size was provided to the participant, and while standing, the length, width and volume were clinically assessed. Participants were then instructed to walk while wearing the shoes and provide feedback pertaining to the level of fit and comfort. If participants were not satisfied with the fit of the shoe, different sizes and/or insole combinations were trialled. Participant-specific information was also taken into account during the fitting process such as use of orthoses and hosiery, anticipated swelling, and personal preference. The final selected size was documented as the ‘allocated shoe size’.

### Perceptions of shoe fit and comfort

While wearing the allocated shoe size, participants were asked to report, on 100 mm visual analogue scales (VAS), their perceptions of shoe fit (using the anchors “poorest fit possible” and “best fit possible”) and comfort (using the anchors “extremely uncomfortable” and “extremely comfortable”).

### 3D foot scanning

The FotoScan 3D foot scanner (Precision 3D Ltd, Weston-super-mare, UK) was used to obtain fully weight bearing scans of both feet. Participants were instructed to stand relaxed with their feet approximately shoulder width apart and their hands clasped lightly in front of them or gently resting on the support rail if required. The FotoScan 3D device uses a fixed system of cameras and projectors to obtain digital images of the foot, which are then automatically converted to 3D models. According to the manufacturer, the scans obtained with this system are accurate to within less than half a millimetre. An interactive example of a scan obtained with this system is provided in Additional file 1.

**Figure 6:**
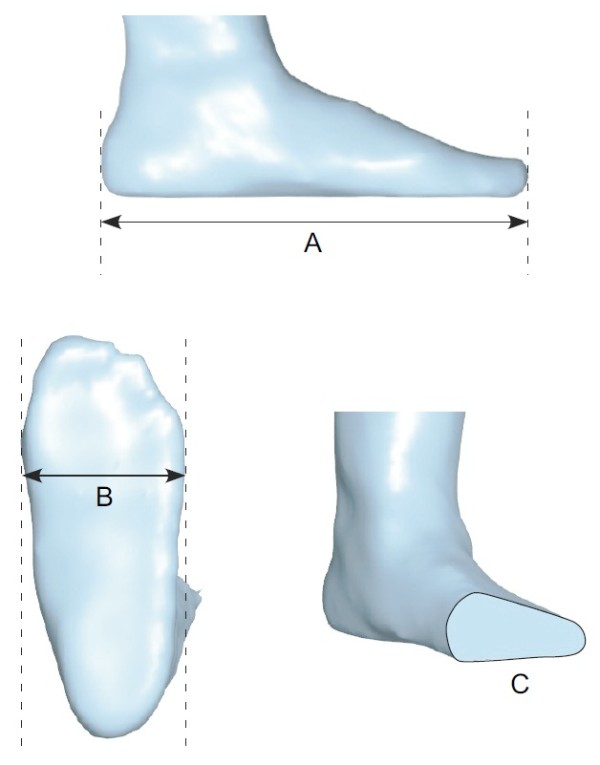
**Foot measurements obtained with the 3D foot scanner. A**: length, **B**: ball width, **C**: ball girth.

The 3D foot scans were exported as stereolithography (STL) files. Using 3D-Tool^©^ Version 10 (3D-Tool GmbH, Weinheim, Germany), foot length and ball width measurements were obtained, and a two-dimensional cross-section of the forefoot (using the widest points of the first and fifth metatarsophalangeal joints) was exported as a drawing exchange format (DXF) file. The total length (perimeter) of this DXF image was determined using Canvas^©^ 11 software (ACD Systems International, Seattle, WA, USA) and documented as ball girth. Figure 6 shows how the measurements were obtained from the scanned image. To evaluate the reliability of the 3D foot scan measurements, 20 randomly selected scans were measured by the same examiner (HBM) on two occasions, one week apart.

### Statistical analysis

All analyses were performed using SPSS version 22.0 (IBM Corp, NY, USA). Although data were collected for both feet, only the right foot data were analyzed in order to satisfy the independence assumption of statistical analysis [26]. To determine the reliability of the 3D foot scanner measurements, intra-class correlation coefficients (ICCs, of the type 3,1) and the percentage coefficient of variation were calculated. To assess the accuracy of shoe fitting, the mean values for foot length, ball width and ball girth obtained from the 3D foot scanner were compared to the corresponding mean values for last length, ball width and ball girth of the allocated shoes provided by the manufacturer using paired-samples *t* tests. Statistical significance was set at the conventional level of α=0.05.

## Results

### Participant characteristics

Postal invitations were sent to 2,457 DVA clients and 341 were screened for eligibility by telephone. Of these, 121 underwent baseline screening and were randomised: 61 into the control group and 60 into the intervention group. Of the 60 participants allocated to the intervention group, two were unable to have their feet scanned due to mobility limitations, one participant’s scan was unusable, and one participant withdrew consent, leaving 56 participants with foot scan data. Characteristics of this sample are provided in Table 1.

**Table 1:** Participant characteristics (n = 56)

Age, mean (SD) years	82.2 (8.0)
Sex, n (%) female	22 (39.3)
Height, mean (SD) cm	165.4 (9.6)
Weight, mean (SD) kg	79.8 (16.9)
Body mass index, mean (SD) kg/m^2^	23.9 (9.7)
Major medical conditions, n (%)	
Diabetes	8 (14.3)
Heart disease	19 (33.9)
High blood pressure	35 (62.5)
Peripheral vascular disease	7 (12.5)
Osteoarthritis	46 (82.1)
Rheumatoid arthritis	3 (5.4)
Stroke	7 (12.5)
Incontinence	14 (25.0)
Use of >4 medications	45 (80.4)
Foot problems	
Foot pain duration, mean (SD) years	17.3 (28.4)
Hallux valgus, n (%)	14 (25.0)
Tailor’s bunion, n (%)	6 (10.7)
Lesser toe deformity, n (%)	42 (75.0)
Dorsal lesions on toes, n (%)	9 (16.1)
Plantar calluses, n (%)	28 (50.0)
Style of footwear worn, n (%)	
Walking shoes	19 (34.0)
Athletic shoes	10 (17.9)
Oxford shoes	10 (17.9)
Moccasin	7 (12.5)
Other	10 (17.9)

### Reliability of 3D foot scanner measurements

The reliability of the 3D foot scan measurements was excellent, with ICCs from 0.964 to 0.999 and CVs from 0.3 to 2.1% (see Table 2).

**Table 2:** Reliability of 3D foot scanner measurements

	**Session 1 mean (95% CI)**	**Session 2 mean (95% CI)**	**ICC (95% CI)**	**CV (%)**
Foot length	251.7 (243.1 to 260.3)	251.7 (243.0 to 260.4)	0.999 (0.996 to 0.999)	0.3
Ball width	92.7 (89.1 to 96.4)	92.0 (88.4 to 95.6)	0.968 (0.920 to 0.987)	1.5
Ball girth	271.4 (258.4 to 284.3)	273.2 (258.8 to 287.5)	0.964 (0.910 to 0.985)	2.1

CI = confidence interval.

ICC = intraclass correlation coefficient.

CV = coefficient of variation.

### Allocated shoe sizes

Allocated shoe size ranged from US size 7 to 14 for men and 5.5 to 11 for women, with 33 (59%) participants requiring an extra width fitting. Thirty-one (55%) participants were allocated a shoe size that differed to the Brannock device®-determined size. Of these, 5 were allocated a shoe that was shorter, 19 were allocated a shoe that was longer, 3 were allocated a shoe that was narrower and 9 were allocated a shoe that was wider. The reasons for the discrepancies between allocated and Brannock device®-determined sizes were as follows: best fit due to discrepancy in size between right and left feet (n = 16), participant preference (n = 9), no half size available (n = 2), best fit due to loose fitting at the ankle (n = 1), larger fit due to anticipation of swelling (n = 1) and accommodation of bulky orthoses (n = 1). Orthosis/insole use was as follows: cushioning insole only (n = 42), flat insole plus own orthosis (n = 9), own orthosis only (n = 3) and cushioning insole plus flat insole (n = 2).

### Comparison between foot dimensions and shoe last dimensions

Table 3 reports the descriptive data (mean, 95% CI and range) for the length, ball width and ball girth dimensions obtained with the 3D foot scanner, the corresponding values from the shoe lasts, and the mean differences between foot and shoe dimensions. The allocated shoes were significantly longer than the foot (mean 23.6 mm, 95% confidence interval [CI] 22.1 to 25.2; *t*_55_ = 30.3, *p* < 0.001), however there were no significant differences in relation to ball width (mean 1.4 mm, 95% CI −0.1 to 2.9 mm; *t*_55_ = 1.9, *p* = 0.066) or ball girth (mean −0.7 mm, 95% CI −6.1 to 4.8 mm; *t*_55_ = −0.2, *p* = 0.810).

**Table 3:** Differences between foot dimensions obtained with 3D foot scanner and corresponding shoe last dimensions

	**3D foot scanner mean (95% CI)**	**Shoe last mean (95% CI)**	Mean difference (95% CI)*	***p *** **value**
Length	255.0 (250.3 to 259.7)	278.7 (273.3 to 284.0)	23.6 (22.1 to 25.2)	<0.001
Ball width	93.8 (92.2 to 95.4)	95.6 (93.6 to 96.7)	1.4 (−0.1 to 2.9)	0.066
Ball girth	260.4 (254.4 to 266.3)	259.7 (255.1 to 264.3)	−0.7 (−6.1 to 4.8)	0.810

CI = confidence interval.

*positive value indicates shoe dimension larger than foot.

### Perceptions of shoe fit and comfort

Participants reported very favourable perceptions of shoe fit, with scores ranging from 66 to 100 mm (mean VAS = 90.7 mm, 95% CI 88.4 to 93.1 mm). Similarly, the shoes were perceived as being very comfortable, with scores ranging from 47 to 100 mm (mean VAS = 88.4 mm, 95% CI 85.0 to 91.8 mm).

## Discussion

The aim of this study was to evaluate the accuracy of shoe fitting in older people who were allocated to the intervention group of a randomised controlled trial assessing the effectiveness of off-the-shelf footwear in reducing foot pain [13]. To do this, we compared the last dimensions of the shoes allocated to each participant to foot dimensions obtained with a high resolution 3D foot scanner. The findings suggest that the fitting protocol used in the study resulted in the selection of appropriately fitting shoes, as evidenced by the shoes being slightly longer than the foot and having equivalent ball width and ball girth measurements. Participants’ overall perceptions of shoe fit and comfort were also very high, providing subjective confirmation of the objective measurements.

Our fitting procedure used a combination of standardised measurement using the Brannock device® and participant feedback to determine the final shoe size allocation. Interestingly, just over half of the sample (55%) was allocated a shoe size that differed to the Brannock device®-determined size. The most common reason for this discrepancy was having a difference between right and left foot sizes, necessitating the selection of a shoe to fit the larger foot. This is consistent with a recent study which reported that just under half of the population has a difference of at least half a US shoe size between left and right feet [4]. However, several other factors contributed to the allocation of a different sized shoe, such as participant preference, accommodation for orthoses and expected foot swelling, suggesting that foot dimensions alone cannot be used to select the optimum shoe size. Indeed, Nácher et al. [27] have shown that a detailed statistical model incorporating 14 anthropometric foot variables was only 66% accurate in identifying the preferred shoe size. Taken together, these findings support the view that fitting shoes is both an art and a science [28] and that optimum shoe size selection needs to take into account a range of factors specific to the individual in addition to accurate measurement of foot dimensions.

On average, the allocated shoes were 23.6 mm longer than the corresponding foot length measurements obtained with the 3D scanner. Although it is widely accepted that shoes need to be slightly longer than the foot to allow for elongation when standing and walking, the recommended distance for the gap between the longest toe and the end of the shoe is essentially arbitrary, varying between 10 to 20 mm (or a ‘thumb’s width’) in the literature [9,22,24,29-32]. The 23.6 mm gap in our study is slightly larger than the upper limit of these recommendations, which suggests that participants may have been allocated shoes that were relatively long. There are three main reasons for this. First, our fitting criteria required that the larger size be allocated to participants with discrepant foot lengths, as no split-sizes were possible within the practical constraints of the study. Second, all participants needed to accommodate some form of insole or orthosis with a contoured heel cup. Third, we allowed for some degree of participant preference in shoe allocation, and five participants requested a shoe size longer than the size determined by the Brannock device®. We consider it unlikely that the slightly longer fitting of the allocated shoes in this study would be detrimental, as shoe discomfort [33] and footwear-related foot disorders [11] are most often related to wearing shoes that are too short rather than too long, and participants reported the shoes to be well fitting and comfortable. However, the 16 week follow-up planned for this study will help to identify whether the fit of the shoes is optimal after longer periods of wear.

There were no significant differences between foot dimensions and allocated shoe dimensions in relation to ball width or ball girth, although there was a trend towards the shoes having slightly larger ball width (mean difference 1.4 mm, *p* = 0.066). This finding needs to be considered in the context of foot deformation when standing and walking, and diurnal variation in foot volume. It has previously been shown that ball width increases by approximately 4% from non-weight bearing to full weight bearing [34-36], and by an additional 3% when walking compared to standing [37]. Furthermore, in healthy older adults, it has been shown that overall foot volume increases by 1.4% after ten minutes of walking [38] and it is likely that older people with venous insufficiency would demonstrate even greater increases in foot volume during the course of a day due to accumulation of oedema [10]. These findings suggest that optimum fitting of shoes requires some allowance for soft tissue expansion, particularly in the forefoot. In our study, the 3D foot scans were taken in a fully weight bearing position, so any soft tissue expansion that occurred from sitting to standing was accounted for. Therefore, only the dimensional changes related to foot loading during gait and oedema would need to be accommodated by the deformation of the upper of the shoe. Given that the shoes used in our trial have a highly pliable Lycra® (elastane) upper, we are confident that the close fit between foot and shoe dimensions in the forefoot represents appropriate fitting. However, this may not have been the case had we selected shoes with less pliable upper materials such as leather.

The findings of this study need to be considered in the context of several limitations. First, we were limited to three variables to characterise the accuracy of shoe fitting. Although several other dimensions can be extracted from 3D foot scans [39], we were only able to obtain length, ball width and ball girth measurements of the shoe lasts from the manufacturer for comparison. These are important parameters, however we acknowledge that a wider array of measurements, such as heel width, instep height, ankle girth and relative lengths of the toes would have provided a more detailed insight into the fit of the shoes [10,40,41]. Second, our assessment of participants’ perceptions of fit and comfort related to the shoe as a whole, and did not delineate the relative fit and comfort of the rearfoot, midfoot and forefoot. Although perceived fit at different foot locations are significantly correlated to each other [32], they are likely to have different implications for the optimum shape and dimensions of the shoe. Third, participants’ reporting of the fit and comfort of the shoes may have been influenced by Rosenthal effects. Fourth, participants in this study were recruited from a veterans’ affairs database, and were required to be ineligible for medical grade footwear to be included. It is therefore possible that older veterans with more pronounced foot deformity were excluded, skewing our sample towards those with more ‘normal’ feet. However, the foot dimensions of participants in our study were very similar to those reported in 668 older people (mean age 64 years) by Chantelau and Gede [9] and 312 community-dwelling older people (mean age 71 years) reported by Mickle et al. [10]. Furthermore, the prevalence of foot disorders in our sample was similar to previous population-based studies of older people [42-44]. This suggests that our findings may be broadly generalisable to the older population, with the exception of those with marked foot deformity who are unable to be accommodated in regular off-the-shelf footwear. Finally, these findings may not be generalisable to other footwear brands, as (i) we used a Brannock device® marked with sizings specific to the Dr Comfort® range, and (ii) the Brannock device® only measures linear ball *width*, so it cannot be assumed that the appropriate fitting of ball *girth* obtained in this study would necessarily translate to other shoes, as the relationship between ball width and girth is not standardised.

In conclusion, this study has shown that shoe size selection in older people using the Brannock device® combined with participant feedback resulted in the allocation of Dr Comfort® shoes with last dimensions that were well matched to the dimensions of the foot determined by a high resolution 3D foot scanner. There are two main implications of these findings. Firstly, in the context of the randomised controlled trial, we can be confident that the protocol used resulted in the provision of appropriately-fitting shoes to the intervention group. However, the longer term follow-up of these participants will assist in determining whether this approach is effective at reducing foot pain. Secondly, in the broader context of clinical practice, our findings suggest that the Brannock device® is a useful clinical tool, but optimum shoe size selection in this age-group may need to take into account a range of factors specific to the individual in addition to accurate measurement of foot dimensions.

## Competing interests

HBM and SEM are Editor-in-Chief and Deputy Editor-in-Chief, respectively, of *Journal of Foot and Ankle Research*. It is journal policy that editors are removed from the peer review and editorial decision-making processes for papers they have co-authored. The remaining authors declare that they have no competing interests.

## Authors’ contributions

HBM and NF conceived the idea and obtained funding for the study. HBM, NF and SEM designed the study protocol. MA and SR collected and entered the data. HBM conducted the statistical analysis and drafted the manuscript. All authors assisted with the writing of the manuscript, and read and approved the final manuscript.

## Supplementary Material

Additional file 1 Example 3D foot scan obtained with the FotoScan 3D foot scanner (Portable Document File). Click here for file
