# Supplementary figures and images for: Evaluation of the accuracy of shoe fitting in older people using three-dimensional foot scanning
Source: J Foot Ankle Res. 2014 Jan 23;7:3. doi: 10.1186/1757-1146-7-3 (PMC3903039; doi:10.1186/1757-1146-7-3)

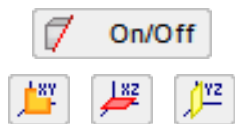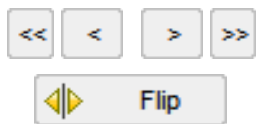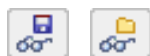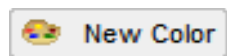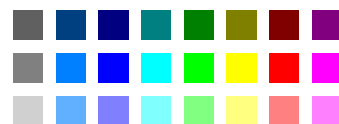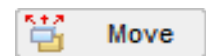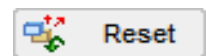

Explode / Move Parts

Supplement: Additional file 1 — Example 3D foot scan obtained with the FotoScan 3D foot scanner (Portable Document File). [file 1757-1146-7-3-S1.pdf]
